# Comparative Analysis of Genome Sequences Covering the Seven *Cronobacter* Species

**DOI:** 10.1371/journal.pone.0049455

**Published:** 2012-11-16

**Authors:** Susan Joseph, Prerak Desai, Yongmei Ji, Craig A. Cummings, Rita Shih, Lovorka Degoricija, Alain Rico, Pius Brzoska, Stephen E. Hamby, Naqash Masood, Sumyya Hariri, Hana Sonbol, Nadia Chuzhanova, Michael McClelland, Manohar R. Furtado, Stephen J. Forsythe

**Affiliations:** 1 Pathogen Research Group, School of Science and Technology, Nottingham Trent University, Nottingham, United Kingdom; 2 Vaccine Research Institute San Diego, San Diego, California, United States of America; 3 Life Technologies Corporation, Foster City, California, United States of America; 4 Life Technologies Corporation, Darmstadt, Germany; 5 Bioinformatics and Biomathematics Group, School of Science and Technology, Nottingham Trent University, Nottingham, United Kingdom; 6 Department of Pathology and Laboratory Medicine, University of California Irvine, Irvine, California, United States of America; Emory University, United States of America

## Abstract

**Background:**

Species of *Cronobacter* are widespread in the environment and are occasional food-borne pathogens associated with serious neonatal diseases, including bacteraemia, meningitis, and necrotising enterocolitis. The genus is composed of seven species: *C. sakazakii, C. malonaticus, C. turicensis, C. dublinensis, C. muytjensii*, *C. universalis*, and *C. condimenti*. Clinical cases are associated with three species, *C. malonaticus*, *C. turicensis* and, in particular, with *C. sakazakii* multilocus sequence type 4. Thus, it is plausible that virulence determinants have evolved in certain lineages.

**Methodology/Principal Findings:**

We generated high quality sequence drafts for eleven *Cronobacter* genomes representing the seven *Cronobacter* species, including an ST4 strain of *C. sakazakii*. Comparative analysis of these genomes together with the two publicly available genomes revealed *Cronobacter* has over 6,000 genes in one or more strains and over 2,000 genes shared by all *Cronobacter*. Considerable variation in the presence of traits such as type six secretion systems, metal resistance (tellurite, copper and silver), and adhesins were found. *C. sakazakii* is unique in the *Cronobacter* genus in encoding genes enabling the utilization of exogenous sialic acid which may have clinical significance. The *C. sakazakii* ST4 strain 701 contained additional genes as compared to other *C. sakazakii* but none of them were known specific virulence-related genes.

**Conclusions/Significance:**

Genome comparison revealed that pair-wise DNA sequence identity varies between 89 and 97% in the seven *Cronobacter* species, and also suggested various degrees of divergence. Sets of universal core genes and accessory genes unique to each strain were identified. These gene sequences can be used for designing genus/species specific detection assays. Genes encoding adhesins, T6SS, and metal resistance genes as well as prophages are found in only subsets of genomes and have contributed considerably to the variation of genomic content. Differences in gene content likely contribute to differences in the clinical and environmental distribution of species and sequence types.

## Introduction

The *Cronobacter* genus (formerly *Enterobacter sakazakii*) is composed of Gram-negative, motile, non-sporeforming, peritrichous rods within the *Enterobacteriaceae* family. This family includes the well-known enteric bacterial pathogens *E. coli* and *Salmonella*, though the *Cronobacter* are most closely related to the *Enterobacter* and *Citrobacter* genera. They are ubiquitous organisms present in a wide range of environments, including water, soil, and a variety of fresh and processed foods [1]. The bacterium has been isolated from factory production lines, including powdered infant formula factories, and households [2] as well as clinical sources such as cerebrospinal fluid, blood, bone marrow, sputum, urine, and faeces [3]. The organism is an opportunistic pathogen of humans that can cause infections in all age groups [4]. However, low birth weight neonates are most at risk and in this host group it has been associated with outbreaks of necrotizing enterocolitis, meningitis, and septicaemia [3], [5], [6], [7], [8], [9]. Infections with these presentations result in exceptionally high mortality rates ranging from 40 to 80 percent [10]. In recent years, several outbreaks of bacterial infection in neonatal intensive care units (NICU) have been traced to powdered formula contaminated with *Cronobacter* spp. [11], [12].

*Cronobacter* was defined as ‘yellow-pigmented *Enterobacter cloacae’* until 1980 when it was designated *Enterobacter sakazakii* by Farmer *et al* [13]. Later analyses of both partial 16S rDNA and *hsp60* sequences showed that *E. sakazakii* isolates formed at least four distinct clusters, and it was proposed that these clusters could be unique species [14]. This was confirmed using DNA-DNA hybridization and phenotyping, and subsequently *Enterobacter sakazakii* was re-classified as the new genus *Cronobacter* [15]. Initially the genus was composed of only four species, but this has been revised and it currently contains the seven species: *Cronobacter sakazakii*, *C. malonaticus, C. turicensis*, *C. muytjensii*, *C. dublinensis*, *C. universalis* and *C. condimenti* [16].

A multilocus sequence typing (MLST) scheme has been established for the entire *Cronobacter* genus and is available online at http://www.pubMLST.org/cronobacter [16], [17], [18]. The scheme is based on seven housekeeping genes (*atpD, fusA, glnS, gltB, gyrB, infB, ppsA*) with a concatenated length of 3036 nucleotides that can be used for multilocus sequence analysis (MLSA). Phylogenetic analysis based on this set of sequences estimated that the separate *Cronobacter* species evolved in the past 40 million years, with *C. sakazakii* and *C. malonaticus* emerging as definable species 11–23 million years ago [18].

The *Cronobacter* MLST scheme has been applied to more than 350 strains that were widely distributed geographically, temporally, and by source, some of which could be traced over a 50-year period. There are currently 121 defined sequence types (ST) covering all *Cronobacter* species, some of which are stable clones [18], [19]. Certain associations have been noted between sequence type and source. For example, *C. sakazakii* ST1 strains are primarily isolates from infant formula and clinical sources, whereas *C. sakazakii* ST8 is primarily composed of isolates from clinical sources. Of special significance is *C. sakazakii* ST4 which appears to have a high propensity for neonatal meningitis [18], [19]. This appears to be a very stable clone as clinical and non-clinical strains have been isolated from 7 countries for over 50 years. In addition, *C. malonaticus* ST7 is associated with adult infections though the source has not been identified.

To date, little is known about the mechanisms of pathogenicity in *Cronobacter*. Candidate virulence determinants include superoxide dismutase (SodA) for macrophage survival [20], haemolysin [21], flagella [22], a metalloprotease [23], an enterotoxin [24], and plasmid-borne virulence factors such as *Cronobacter* plasminogen activator (Cpa) and type six secretion systems (T6SS) [25]. The bacteria can attach to intestinal cells and survive in macrophages [20], [26]. OmpA and OmpX possibly have a role in the organism penetrating the blood-brain barrier, though the mechanism leading to the destruction of the brain cells is unknown and could, in part, be a host response [27]. However, only a few studies have described the interaction of *Cronobacter* spp. with human cells, and the specific receptors involved remain to be determined. It is known that the disruption of tight junctions significantly enhances association of *C. sakazakii* with the human intestinal cell line Caco-2 [28]. Strains from *C. sakazakii* and *C. malonaticus* show higher invasion of Caco-2 than other *Cronobacter* species [20]. Similarly, both *C. sakazakii* and *C. malonaticus* survive and replicate in macrophages inside phagosomes, whereas *C. muytjensii* dies and *C. dublinensis* is serum sensitive. Virulence also varies within the *C. sakazakii* species as evident from epidemiological studies of a NICU outbreak in France where the clinical outcome of three *C. sakazakii* pulsetypes differed, with only one pulsetype (now recognised as ST4 strains) causing the three deaths [8], [19]. To date, only strains from *C. sakazakii, C. malonaticus* and *C. turicensis* have been associated with reported neonatal infections, whereas *C. malonaticus* ST7 strains were associated with adult infections [19]. Therefore pathogenicity in humans may be an acquired trait in this genus.

Kucerova et al. [3], [29] used whole genome sequence analysis of *C. sakazakii* strain BAA-894 and microarray based comparative genomic hybridisation (CGH) to explore the genetic basis of virulence in *Cronobacter*. CGH highlighted 15 clusters of genes in *C. sakazakii* BAA-894 that were divergent or absent in more than half of the tested strains; six of these were of probable prophage origin. Several putative virulence factors (i.e. fimbriae and multidrug efflux systems) were identified in these variable regions.

Here we present a comparative genomic sequence analysis of eleven recently sequenced genomes from the seven *Cronobacter* species fully representing the genus, and including the recently recognised species *C. universalis* and *C. condimenti* [16]. These strains were chosen as representatives of the *Cronobacter* genus based on seven locus MLSA [17], [18]. The three *C. sakazakii* strains (680, 696 and 701) are well characterised clinical isolates previously reported in eight publications [3], [8], [17], [18], [19], [20], [29], [30]. In addition, strain 701 is a *C. sakazakii* ST4 strain that was isolated from a fatal case of neonatal meningitis and has been used for *in vitro* tissue culture studies of virulence [8], [26]. *C. sakazakii* 680 is ST8, a sequence type associated with clinical infections without being linked to infant formula consumption [17], [19]. *C. malonaticus* 681^T^ is ST7, a sequence type associated with adult infections. According to MLSA, *C. condimenti* is distantly related to the other *Cronobacter* species [18]. Completed genomes of *C. sakazakii* BAA-894 and *C. turicensis* z3032 were already in the public domain [29], [32] and were used to accurately match reads to reference sequences. The incomplete draft genome of *C. sakazakii* E899 [32] became available during the completion of this study and was used as a comparator where appropriate. Thus, a total of 14 genomes were used in this comparative study.

The aim of this study was to determine (i) the core *Cronobacter* genome, (ii) specific regions related to physiological and virulence related traits, and (iii) unique regions in *C. sakazakii* ST4, associated with neonatal meningitis [19], [30], and in *C. malonaticus* ST7 associated with adult infections. Due to the severity of infant infection, a better understanding of the genomic variation between *Cronobacter* spp. is needed, and will be of interest to manufacturers of powdered infant formula, regulatory bodies, and those studying the evolution and diversity of bacterial pathogenicity.

## Results and Discussion

### *Cronobacter* Genomes

Using the SOLiD® 4 System and Ion Torrent PGM™ next generation sequencing platforms, we sequenced the genomes of eleven *Cronobacter* strains including three *C. sakazakii*, two *C. malonaticus*, one *C. muytjensii*, one *C. turicensis*, two *C. dublinensis*, one *C. universalis*, and one *C. condimenti* (Table 1). The sum of the contig lengths ranged from 4.4 to 4.9 Mb, in length, comparable to the genomes sizes of the previously sequenced *C. sakazakii* BAA-894 and *C. turicensis* z3032 genomes [29], [31]. The publicly available, incomplete genome of *C. sakazakii* E899 is only 3.96 Mb and appeared to lack plasmid sequences [32]. Genome comparison revealed that pair-wise DNA sequence identity varies between 89 and 97%. A BRIG alignment for all the 14 *Cronobacter* genomes using the *C. sakazakii* BAA-894 genome as a backbone is shown in Figure S1.

**Table 1:** Eleven newly assembled and three publicly available *Cronobacter* genomes analyzed in this study.

Species	Strain	Source	Country	Genome size (Mbp)^d^	MLST Sequence Type	EBI Accession Number
*C. sakazakii*	680	Clinical	USA	4.36	8	CALG01000001-CALG01000201
	696	Clinical	France	4.90	12	CALF01000001-CALF01000569
	701	Clinical	France	4.75	4	CALE01000001-CALE01000768
	E899^a^	Clinical	USA	3.96	4	AFMO00000000
	BAA-894^a^	Powdered formula	USA	4.53	1	NC_009778-NC_009780
*C. malonaticus*	507	Clinical	Czech Republic	4.45	11	CALD01000001-CALD01000249
	681^b^	Clinical	USA	4.50	7	CALC01000001-CALC01000171
*C. turicensis*	564	Clinical	USA	4.50	5	CALB01000001-CALB01000114
	z3032a,c	Clinical	Switzerland	4.60	19	NC_013282-NC_013285
*C. dublinensis*	582	Unknown	UK	4.68	36	CALA01000001-CALA01000427
	1210^d^	Environment	Ireland	4.59	106	CAKZ01000001-CAKZ01000221
*C. muytjensii*	530	Infant formula	Denmark	4.53	49	CAKY01000001-CAKY01000365
*C. universalis*	NCTC 9529^T^	Environmental	UK	4.45	54	CAKX01000001-CAKX01000231
*C. condimenti*	1330^e^	Food	Slovakia	4.46	98	CAKW01000001-CAKW01000155

a Strains for which genome sequence is publicly available; E899 appears to lack plasmid sequences.

b *C. malonaticus* species type strain (LMG 23826^T^).

c *C. turicensis* species type strain (LMG 23827^T^).

d *C. dublinensis* species type strain (LMG 23823^T^).

e *C. condimenti* species type strain (LMG 26250^T^).

f Sizes of newly sequenced genomes were derived from the sum of the length of all contigs from *de novo* assembly.

### Phylogenetic and Evolutionary Analysis of the *Cronobacter* Genus

**Figure 1:**
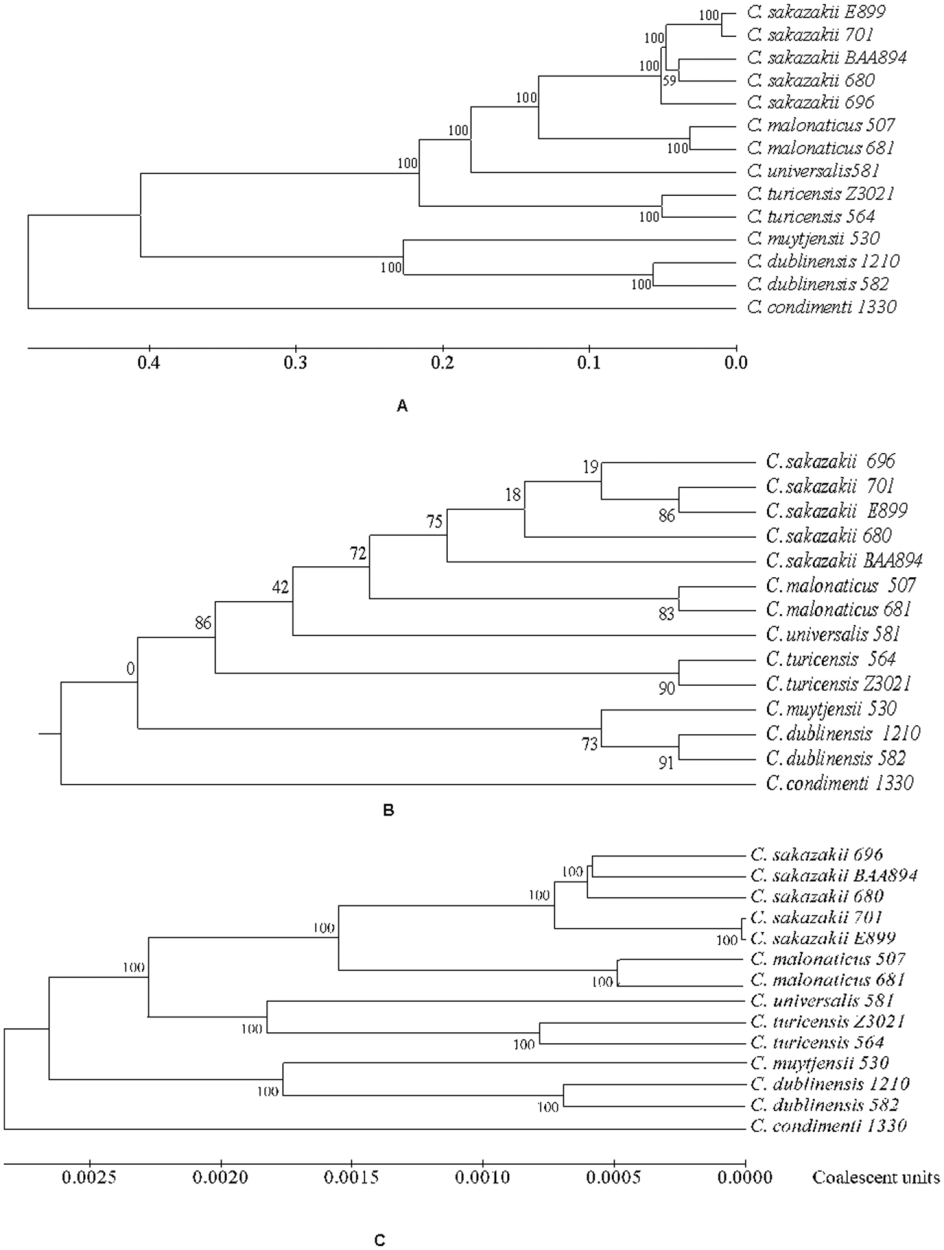
Phylogentic analysis of *Cronobacter* spp. based on whole genome sequencing. (A) A maximum likelihood phylogram based on 138,143 SNPs identified from a concatenated alignment of 1117 core genes (879,768 nucleotides) present in single copies in all strains. The numbers on the nodes represent the bootstrap support from 1000 replicates (in percentage). (B) A majority rule consensus tree generated by summarizing individual 1117 core gene trees. The numbers on the internal nodes indicate the fraction (in percentage) of gene trees which support the partition of the taxa into the two sets. (C) A Clonal Frame (CF) analysis based on randomly selected 99 core loci. The consensus tree was obtained from 2 CF runs with 200,000 MCMC iterations. The numbers on the nodes represent the bootstrap support in percentage.

**Figure 2:**
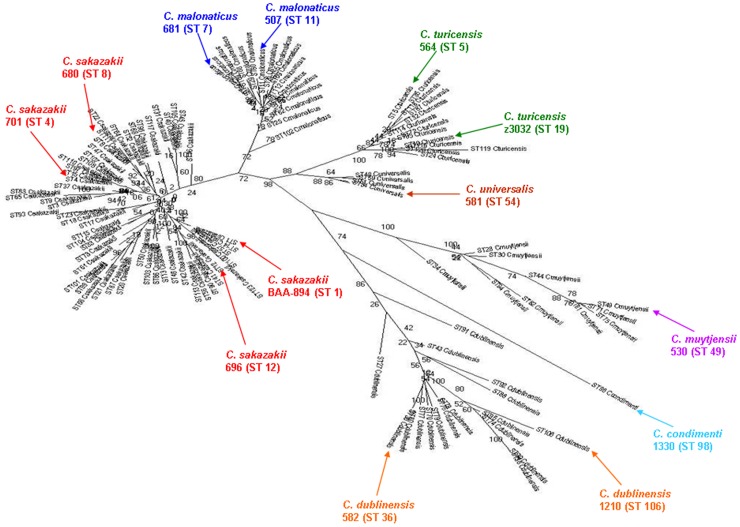
Maximum Likelihood tree based on the concatenated sequences (3,036 bp) of the seven MLST loci. The tree is drawn to scale using MEGA, with 1000 bootstrap replicates.

Figure 1A represents a maximum likelihood (ML) phylogram constructed based on the 138,143 SNPs identified from a concatenated alignment of 1117 core genes (879,768 nucleotides) present in single copies in all strains. Figure 1B represents a majority rule consensus tree generated by summarizing all the individual gene trees. The topology of speciation for each *Cronobacter* species was similar, as predicted by the two different approaches. There were contradictions in resolving the nodes within *C. sakazakii* indicating that recombination events might have obscured the phylogenetic signals within individual gene trees. Within *C. sakazakii* there was a higher level of confidence in placing *C. sakazakii* 701 and *C. sakazakii* E899 within the same clade, indicating that these two strains might have evolved more recently from a common ancestor. MLST revealed these two strains are both ST4 (see Figure 2 for MLSA phylogenetic analysis of the fourteen sequenced). Figure 1C represents the results of Clonal Frame analysis [33] of 99 randomly chosen core loci. Clonal Frame tries to recreate the evolutionary descent of each sequence by taking into account the recombination events that may have occurred during its evolutionary history. Clonal Frame also compares the recombination and mutation rates in assessing the impact of recombination on the evolution of observed phylogeny. Consensus tree (Figure 1C) obtained from two Clonal Frame runs with 200,000 MCMC iterations had a topology similar to the ML tree obtained using core genome SNPs, with one important difference. Clonal Frame analysis predicted that *C. universalis* and *C. turicensis* directly evolved from a common ancestor, while the ML tree of core SNPs (Figure 1A) predicted that they evolved in a step-wise fashion. This correlates well with the phylogenetic analysis of the seven loci used in the *Cronobacter* MLST scheme [18]. Co-incidentally, among all the speciating nodes in the consensus tree of 1117 core genes, the node where *C. universalis* speciates has the most incongruence. Hence, based on these two observations, it can be inferred that after speciation there might have been a large scale recombination event between ancestral *C. universalis* and hitherto unknown source. Clonal Frame predicted that for the observed phylogeny the ratio of substitutions introduced by recombination as compared to mutation (*r*/*m*) was 0.32, while ratio of number of recombination events to mutation events (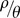) was 0.01.

Comparisons between the evolutionary rates of gene families did not reveal a clear variation between the species of the *Cronobacter* genus. However, an enrichment analysis performed to assess the statistical over-representation of relatively fast evolving genes within certain functional categories revealed that genes related to iron acquisition, copper homeostasis, phage tail proteins, and genes related to O-antigen and fimbriae evolved faster as compared to the rest of the *Cronobacter* pan genome.

### Core and Pan Genome Identification

A pan genome analysis of the fourteen genomes of the *Cronobacter* genus was used to study the diversity of the genomes, to identify genes of phenotypic and pathogenicity interest and those unique to each species. Putative prophage regions, identified using Prophinder [34], were included in the analysis. Some of the characteristics of the genomes of each of the species are discussed below. Additional detail is given in Text S1.

**Figure 3:**
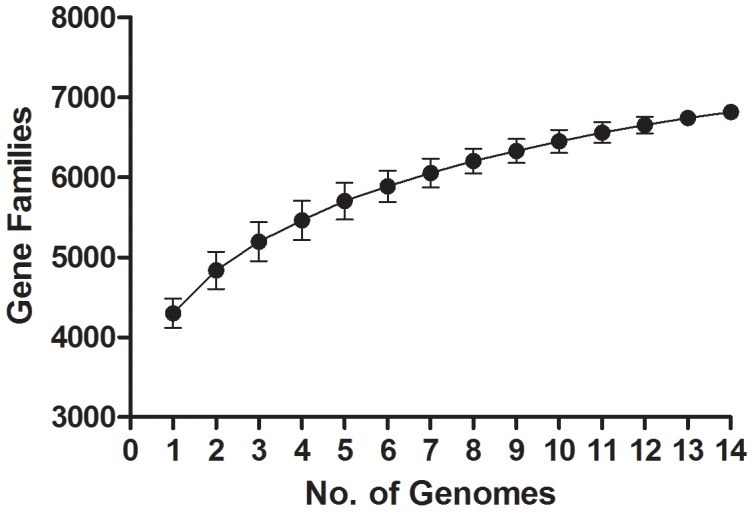
Graph representing the rarefaction of the *Cronobacter* pan genome, generated using the 14 *Cronobacter* genomes included in this study.

All genomes were annotated using RAST [35]. The number of annotated genes per genome varied between 3,700 and 4,200. Using the binomial mixture model approach of Snipen et al. [36], the observed pan genome was estimated to contains 6,155 genes, of which 2,271 (36.9%) genes were conserved in all fourteen *Cronobacter* genomes, an estimate of the core genome, and 3,884 (63.1%) were accessory genes found in at least one of the fourteen genomes studied. Because the draft genomes can have up to thousands of sequence gaps, some genes may have incomplete fragments or were unannotated. This could cause a slight under-estimation of the size of the core genome and a slight over-estimation of the size of the pan genome. A graph representing the rarefaction of the *Cronobacter* pan genome has been generated and is shown in Figure 3.

The published genome of *C. sakazakii* BAA-894 contains two plasmids pESA2 (31 kb, 51% GC content) and pESA3 (131 kb, 56% GC), encoding for 38 and 127 genes respectively. In contrast, *C. turciensis* z3032 contains three plasmids pCTU2 (22.5 kb, 49%GC), pCTU3 (53.8 kb, 50% GC) and pCTU1 (138 kb, 56% GC), encoding for 32, 74 and 136 genes respectively. The larger plasmids, pESA3 and pCTU1, share a common backbone and genes encoding for potential virulence traits such as iron acquisition systems (*eitCBAD* and *iucABCD/iutA*). The differences include the presence of plasminogen activator gene (*cpa*) and T6SS in the pESA3 plasmid, whereas in pCTU1 there is a 27 kb region with homologies to *fhaB*, *fhaC* and five adhesins (FHA locus) [25]. The *cpa* gene was only found in *C. sakazakii* strains 696 and 701, and was absent from 680. It was also absent from all other *Cronobacter* species with the exception of *C. universalis*. The BRIG alignments with pESA3 and pCTU1 as reference backbones are shown in Figures S2 and S3, respectively. It can be seen that the plasmid backbone was conserved in all the strains with varying degrees of homology. There were also clear regions of variation between the pESA3 and pCTU1 plasmids, some of which have been previously reported. However, it should be noted that matches to the plasmid do not necessarily confirm their location. To address this issue the read coverage was used to predict the location of the plasmid-borne genes. Genes homologous with those in pESA3 and pCTU1 were found in all sequenced strains of all species, though pESA3 was only partial in *C. sakazakii* 680 and *C. condimenti* 1330^T^, and pCTU1 had a partial match in *C. sakazakii* 680, 696, 701, and *C. condimenti* 1330^T^.

Previously, Kucerova et al. [29] detailed three prophages and 3 partial prophages in *C. sakazakii* BAA-894 contributing to the genome diversity; none of these prophages were previously reported in *C. turicensis* z3032 [31]. In our study of the whole genus we found a total of 27 major prophage regions (>20 ORFs) across the 13 genomes, most of which were found to be shared between the *Cronobacter* species (Table S1). In addition, we found a clustered regularly interspaced short palindromic repeats (CRISPR) array, which was located in the region ESA_02830-38. CRISPR arrays consist of a series of highly conserved repeats ∼20–50 bp separated by unique DNA fragments (spacers) of a similar length. It is understood that the CRISPR system provides a mechanism of resistance to infection by phage to which the bacteria have previously been exposed. Various CRISPR-associated (*cas*) genes and specific adjacent sequences are required to trigger these processes [37]. This specific CRISPR region was present in all *Cronobacter* strains except *C. sakazakii* 680 (ST8).

Comparison of the three *C. sakazakii* genomes revealed 408 ORFs, which were unique within this species. A number of regions were found to be unique to the genomes of strains 696 (ST12) and 701 (ST4). The *C. sakazakii* 680 (ST8) genome had unique genes encoding a region comprising iron uptake (*fecRABCDE*), a *lac* operon, and arsenate resistance on the larger plasmid. It also had large regions missing compared with the reference genome *C.* *sakazakii* BAA-894, including flagella synthesis (ESA_01224-69) and CRISPR (ESA_02831-37). The two *C. malonaticus* genomes revealed 92 ORFs unique to the species. Only about 15% of these ORFs were conserved between the two genomes. The *C.* *malonaticus* 681^T^ (ST7) unique ORFs included mainly phage related proteins and two genes related to capsular polysaccharide export proteins. The *C. turicensis* 564 genome was analysed along with the publicly available *C. turicensis* z3032 genome to reveal 137 ORFs unique to the species, of which less than 10% were shared between the two genomes. Comparative analysis of *C. dublinensis* genomes revealed 142 ORFs unique to the species, of which less than 20% were shared between the two genomes. The *C. dublinensis* 582 genome also showed the presence of two additional clusters of CRISPR (938–943 and 3962–68). Only single strains were sequenced for each of the remaining *Cronobacter* species. The *C. universalis* genome had 73 unique ORFs. This included a region of 36 ORFs, which appears to be a plasmid remnant encoding for conjugation (*trb* genes), replication initiation, and plasmid stabilization. There were 40 ORFs found to be unique to the genome of *C. muytjensii* strain 530. The genome of the newly defined species *C. condimenti* strain 1330^T^ contained 79 unique ORFs. These included genes related to β-xylosidase and a cluster of eight genes that are indicative of a unique fucose biosynthesis pathway.

### Physiological and Phenotypic Traits in the *Cronobacter* Core and Pan Genome

The presence of known genes conferring physiological and phenotypic traits, categorized according to environmental stress response, cell surface composition, sugar metabolism, and metal resistance was examined.

A number of key stress response genes were investigated. A gene homologous to the universal stress protein *uspA* (ESA_01955) was detected in all strains. Similarly, homologues were located for stringent starvation response (ESA_03615), carbon starvation sensing protein *rspA* (ESA_01752), and carbon starvation protein (ESA_00801) in all *Cronobacter* strains. However, an additional carbon starvation homologue (ESA_00339) was found in all *Cronobacter* strains except *C. sakazakii* 680 due to the long missing region mentioned above. *Cronobacter* is known for its ability to survive desiccation for up to two years [38], [39]. Genes involved in desiccation resistance and osmotic stress adaption were found in all *Cronobacter* species. This included genes encoding the uptake of the osmoprotectants glycine, betaine, and trehalose (ESA_00587-9, ESA_01944-66), which were present in all *Cronobacter*. Thermal tolerance in *Cronobacter* has been controversial due to conflicting reports [40]–[42]. The initiation translation factor (*infB*) proposed by Asakura et al. [41] was present in all *Cronobacter*, and is one of the alleles used for MLST. In contrast, the thermoresistance gene cluster (or most components) identified by [42] was only present in *C. sakazakii* strains 696, 701, and in both strains of *C. malonaticus*. Unfortunately, this region is poorly annotated and the association with thermal resistance is not supported by experimental data in these strains. Together with biofilm formation, the ability to adapt and persist under stressed conditions enables the bacterium to survive in dry food ingredients. All *Cronobacter* species had genes (*crtZ*; ESA_00341-4) for β-carotene production, a yellow pigmentation, which we speculate helps protect the organism from light produced by oxygen radicals.

Capsular polysaccharides on the bacterial cell surface can play a crucial role in the interaction of bacteria with their environment and in their pathogenicity. The genes encoding the enzymes responsible for the production and transport of these polysaccharides are usually clustered in large operons, such as the *wca* operon in *E. coli* K-12. This gene cluster controls the biosynthesis of the exopolysaccharide colanic acid containing fucose and glucuronic acid. *Cronobacter* spp. can produce copious extracellular polymers, resulting in highly mucoid colony formation and can form biofilms on inert surfaces including infant feeding equipment and nasogastric feeding tubes [8], [43], [44]. Colanic acid production was encoded (ESA_01155-01175; *wzABCKM*) in all sequenced *Cronobacter* strains. The genes for capsular polysaccharide assembly and export (ESA_03350-59) were present in all *Cronobacter* strains except for two clinical isolates, *C. sakazakii* 701 (ST4) and 696 (ST12).

One highly variable genomic region (ESA_01179–89) corresponds to the O-antigen gene locus. The locus contains two genes *galF* (ESA_01177) and *rfbB* (ESA_01178) which are conserved in all the *Cronobacter* strains, whereas the rest of the genes from the O-antigen locus are highly divergent and were not detected previously by microarray hybridization [29]. The lipopolysaccharide (LPS) is one of the few structural features of *Cronobacter* which has been chemically investigated and is known to vary across the genus. In *C. sakazakii* and *C. malonaticus* the LPS are composed of various branched polymers, whereas they are unbranched in *C. muytjensii* [45]–[48].

Variation in the O-antigen region has been used in serotype-specific PCR-based assays [49]–[53]. The regions between UDP-glucose pyrophosporylase (*galF*) and 6-phosphogluconate (*gnd*) were amplified to generate distinguishable O-antigen groups. The genetic architecture of the O-antigen cluster in the reference genome *C. sakazakii* BAA-894 (ST1) corresponds to the serotype O:1 and both the ST4 strains 767 and NCTC 8155 are O:2. However, the current PCR-based serotyping assay has a significant limitation since some defined serotypes are found in more than one species [51]–[53].

It is notable that *C. sakazakii* 701 (ST4) also encoded an O-antigen acetylase (O-acetyl transferase; 28141.13.peg.3410) adjacent to a prophage region which, except for fragments in *C. sakazakii* 696 (ST12), was not present in other *C. sakazakii* strains. O-antigen modification enzymes are encoded by temperate bacteriophages [54]. The possibility of this in *Cronobacter* was proposed by Sun et al. [51] when comparing RFLP patterns from PCR amplification of the O-antigen genes of 119 *C. sakazakii* strains. Seroconversion is an important virulence factor since antigenic variation enhances the survival of the bacteria because the host would have to mount a specific immune response to each different serotype. Serotype-converting bacteriophages play an important role in conferring these traits, and recognising their presence in *C. sakazakii* is important due to the application of the serotyping scheme.

The gene cluster encoding for maltose utilisation and including α–glucosidase (ESA_02709-14) was found in all *Cronobacter* strains. The presence of α-glucosidase and a constitutive maltose uptake system, as well as osmotic pressure tolerance, has been used as the basis for a number of commercially available enrichment broths and chromogenic agars for the isolation of *Cronobacter* spp. [55]. Other sugar utilization pathways were only found in some *Cronobacter* species. A gene cluster for α-mannosidase utilisation (ESA_02616-18) was only found in *C. sakazakii* and *C. turicensis,* and not in the closely related *C. malonaticus*.

*Cronobacter* are ubiquitous in the environment, and their main habitat is believed to be plant material. *Cronobacter* are able to ferment β-glucoside sugar substrates from plants, including cellobiose, arbutin, salicin, and esculin. The genes involved in the metabolism of β-glucosides are in the cluster ESA_02544-47. ESA_02544 is probably a transcriptional antiterminator from the BglG family, which is involved in positive control of the utilization of different sugars by transcription antitermination [56]. ESA_02545 encodes a kinase which converts β-glucosides to 6-phospho-β-glucosides and ESA_02546 encodes a 6-phospho-β-glucosidase specific to arbutin-6-phosphate and salicilin-6-phosphate. ESA_02547 also encodes 6-phospho-β-glucosidase, which may have the same or similar function as ESA_02546. Malonate is also found in plants and *C. malonaticus* was initially a subspecies within *C. sakazakii* that was differentiated from *C. sakazakii* subsp. *sakazakii* by the malonate utilisation test [15]. However, the genes required for malonate metabolism - malonyl CoA acyl carrier protein transacylase, phosphoribosyl-dephospho-CoA transferase, and malonate decarboxylase (Ctu_34990-35070) - are found in all *Cronobacter* species except *C. sakazakii* and *C. dublinensis* strain 582. Therefore this test cannot be used solely for the purpose of identifying *C. malonaticus*.

Homologues of tellurite resistance genes (*terACDYZ*) were located in the loci ESA_01775–ESA_01804 of *C. sakazakii* BAA-894. As the gene cluster was absent from all other *Cronobacter* strains studied, the reference strain BAA-894 or the ST1 lineage probably acquired the tellurite resistance cluster recently. Homologies of these genes are also found on the IncII plasmid R478 of *Serratia marcescens,* pK29 of *Klebsiella pneumoniae* NK29, pEC-IMPQ of *Enterobacter cloacae*, and pAPEC-O1-R of *E. coli* APEC O1 [57]. In BAA-894 this cluster of genes contains an IS element (ISEhe3) just before ESA_01781 which was also identified on the pK29 plasmid as well as a fragment of an IS4 family transposase within the locus ESA_01803.

Copper is essential for bacterial growth, being required for many key enzymes, but is toxic in excess. Two copper and silver resistance gene clusters are located close to each other (ESA_04238-45 and ESA_04248–55) in the *C. sakazakii* BAA-894 annotated chromosome and are on the smallest plasmid (pCTU3) in *C. turicensis* z3032 (pCTU3_3p00600-700 and pCTU3_3p00490-590). The first region (*cusRCFBA/silRECBA*) was present in all *C. sakazakii* strains, in one *C. malonaticus* (681) strain, and one *C. turicensis* (z3032) strain. CusA is a membrane protein belonging to the resistance-nodulation-division (RND) protein superfamily. CusF is a periplasmic protein, which in *E. coli* interacts with the cusCBA efflux system. The second region (*pcoABCDR*) was present in nearly all *C. sakazakii* strains (except 701), in one *C. malonaticus* strain (681), one *C. turicensis* strain (z3032), and in *C. universalis* 581^T^. PcoA encodes a homologue to multicopper oxidase, and PcoB is an outer membrane protein transfering copper into the periplasm where it is oxidised by PcoA. Both these regions are encoded on the plasmids R478 and APEC-O1-R, referred to above, which carry tellurite resistance genes [57]. The copper and silver resistance genes on the reference *C.* *sakazkaii* BAA-894 genome are separate from the tellurite resistance genes. Therefore these regions do not appear to be the result of a single plasmid integration event.

### Virulence Associated Genes

Putative virulence traits in *Cronobacter* include (i) attachment and invasion of host cells, (ii) iron acquisition, (iii) macrophage survival, and (iv) protein secretion systems. Since genes encoding for SodA, OmpA, OmpX, and a metalloprotease (*zpx*) were found in all genomes irrespective of source and species, they could not be connected to particular virulence-associated strains such as *C.* *sakazakii* ST4 and therefore to neonatal meningitis.

*Cronobacter* spp. route of infection is probably through attachment and invasion of the intestinal cells and therefore genes encoding surface appendages such as fimbriae (pili) are of importance. The genetic content of all fimbriae clusters was most similar to the type I chaperone/usher-assembled pilus system [58]. These clusters may encode complete and functional pili, as some degree of homology was found between the genes in the *C.* *sakazakii* fimbriae clusters and the remaining components necessary for type-I pilus assembly (the minor tip fibrillum *fimG* and fimbrial adhesin *fimH* genes). Type 1 fimbriae have been associated with *E. coli* K1 invasion of human brain cells [59] and are therefore of particular interest in *Cronobacter* pathogenicity studies.

A total of ten putative fimbriae gene clusters were identified and are summarised in Table 2. Curli fimbriae are believed to play an important role in the adhesion of *E. coli* to host cells by interacting with matrix proteins such as fibronectin, laminin, and plasminogen to initiate adherence and colonization. However, since *C. sakazakii* strains dominate clinical isolates, the absence of curli fimbriae genes infers this trait is not essential for *Cronobacter* pathogenicity. Why the region has been lost in the *C. sakazakii* lineage and retained in the rest of the genus is unclear.

**Table 2:** *Cronobacter* fimbriae cluster designations.

Cluster	Loci	*C. sakazakii*				*C. malonaticus*		*C. muytjensii*	*C. turicensis*	*C. dublinensis*		*C. universalis*	*C. condimenti*	Comment
		BAA-894	680	696	701	507	681	530	564	582	1210	NCTC 9529^T^	1330	
1	ESA_01970-76	+	+	−	−	−	−	−	−	−	−	−	−	Also found in *C. sakazakii* strain2 [Kucerova et al. 2010; 35]
2	ESA_02342-45	+	+	+	+	+	+	+	+	+	+	+	+	Present in all *Cronobacter*
3	ESA_02538-42	+	+	+	+	+	+	+	+	+	+	+	+	ESA_02541&42 not found in *C. muytjensii*, *C. dublinensis*, *C. condimenti*
4	ESA_02795-99	+	+	+	+	+	+	−	−	−	−	−	−	Only found in *C. sakazakii* and *C. malonaticus*
5	ESA_03512-20	+	+	+	+	−	−	−	−	−	−	−	−	Beta-fimbriae. Only found in *C. sakazakii*
6	ESA_04067-73	+	+	+	+	+	+	+	+	+	+	+	+	Present in all *Cronobacter*
7	ESA_03812-15	+	+	+	+	+	+	−	+	+	+	+	+	Absent from *C. muytjensii*
8	Ctu_36390-450	−	+	+	+	+	+	+	+	+	+	+	+	π–fimbriae. Absent from *C. sakazakii* BAA-894
9	ESA_03231-33	+	+	+	+	+	+	+	+	+	+	+	+	Type IV. Gliding motility
10	Ctu-16160-230	−	−	−	−	+	+	+	+	+	+	+	+	Curli fimbriae. Absent from *C. sakazakii*

Iron is an essential growth factor and bacteria have evolved mechanisms, such as siderophores, for its acquisition. Nearly all *Cronobacter* strains examined possess complete operons for the production of enterobactin synthesis (*entABCDEFS*, ESA_00791-800), receptor, and transport (*fepABCDEG*; ESA_02727-31). The exception were *C. sakazakii* strains 701 and 696, which lacked the homologue for EntS, an enterobacterin exporter. All *Cronobacter* species possess a complete plasmid-borne operon for aerobactin synthesis (*iucABCD*) and its receptor *iutA* (ESA_pESA3p05547-51). Additionally, a gene cluster for hydroxamate-type siderophores (*fhuABCDE*, ESA_03187-90 & ESA_02242) was encoded in all *Cronobacter* species. As previously referred to, *C. sakazakii* 680 encoded a unique siderophore (*fecRABCDE*) which is probably plasmid-borne. According to Franco et al. [25], the only functional siderophores are those encoded on the larger plasmid. Iron uptake is often cited as a virulence mechanism, especially with respect to plausible utilisation by *Cronobacter* of iron in breast milk and infant formula. However, drawing further conclusions will require further work because there are a number of probable iron uptake mechanisms across the genus, including those in species not associated with neonatal infections: *C. muytjensii*, *C. malonaticus*, *C. dublinensis*, *C. universalis*, and *C. condimenti*.

The secretion systems ancestrally related to the bacterial conjugation machinery are referred to as the type IV secretion systems (T4SSs). The T4SSs can transfer both proteins and nucleoprotein complexes and could constitute a conjugal transfer system [60]. A T4SS, unique to *C. sakazakii* BAA-894 and *C. turicensis* z3032, was located on the smaller plasmid (pESA2 31kB and pCTU2 22.5 kB) of both strains. A second cluster of 19 genes, belonging to the *Tra* group of genes and annotated as IncF conjugation plasmids, was found to be present in the *C. sakazakii* 701 and 696 genomes and is linked to the type IV protein secretion system. This could also be a plasmid-borne region as these genes have previously been identified in the Inc group of plasmids in *E. coli* and *Serratia.*

Type VI secretion system (T6SS) is a newly described system that may be involved in competing with other bacteria in adherence, cytotoxicity, host-cell invasion, growth inside macrophages, and survival within the host. One T6SS associated gene that was located separately from the main T6SS gene cluster, was *vgrG*, encoding a lipoprotein (ESA_00292-4). It was present in all *Cronobacter* strains except for one strain of *C. malonaticus* (507) and one strain of *C. muytjensii* (530). Six putative T6SS clusters were identified in the *Cronobacter* genomes (Table S2), some of which were previously described by Kucerova et al. [3], [29]. These are putative T6SS clusters as it remains to be determined whether they encode functional secretion systems. Zhou et al. [61] recently reported the role of T6SS in *E. coli* K1 invasion of the human blood-brain barrier. Although several T6SS are found in all *Cronobacter* species, none were unique to *C. sakazakii* ST4 strain 701.

All sequenced *Cronobacter* genomes exhibited the presence of various hemolysin and hemolysin-related genes, scattered across the genome (Table S3). These genes were present in all the genomes with the only exceptions being *C. sakazakii* 701 and *C. malonaticus* 507, which lacked the 21 kDa hemolysin precursor gene. Most of the strains had two copies of the hemolysin gene and the hemolysin activator protein precursor gene.

One of the most interesting gene clusters, unique to *C. sakazakii* is ESA_03609–13 (*nanKTAR*) and ESA_03302 (*nanC*) which encode for the uptake and utilization of exogenous sialic acid, which is functional (unpublished laboratory studies). Sialic acid catabolism is limited to commensal bacteria in the intestinal tract and to pathogens [62]. In *C. sakazakii* ESA_03609 encodes a putative sugar isomerase (YhcH). Genes ESA_03610-13 encode the *nanKTAR* genes involved in the N-acetylneuraminate and N-acetylmannosamine degradation pathway. The *nanK* gene (ESA_03610) encodes N-acetylmannosamine kinase; *nanT* (ESA_03611) encodes the sialic acid transporter N-acetylneuraminate lyase; *nanA* (ESA_03612) encodes N-acetylneuraminate lyase, and *nanR* (ESA_03613) is a transcriptional regulator from the GntR family. The *nanE* locus encoding the enzyme N-acetylmannosamine-6-phosphate-2epimerase was located separate from this cluster at ESA_00529, and unlike the rest was found conserved across the genome of the *Cronobacte*r genus. *NanC* (ESA_03302) encodes the N-acetylneuraminic acid outer membrane channel protein). It is notable that the *nan* cluster is adjacent to a stringent starvation gene homologue (*sspA*, ESA_03615) and therefore expression of the *nan* cluster could be responsive to environmental nutrient levels. The acquisition of genes encoding for the utilization of exogenous sialic acid may have a major role in *C. sakazakii* colonisation of the human intestinal tract (via mucins) and the use of sialic acid in breast milk, infant formula, and brain cells as a nutrient source [62].

Despite detailed genomic analysis, the reason for the association of *C. sakazakii* ST4 with neonatal meningitis remains unclear. Initially Joseph & Forsythe [19] proposed that it could be either environmental persistence, resulting in greater neonatal exposure, or the presence of specific pathogenicity traits. Other than one putative plasmid-borne T6SS, this study has not shown any conclusive unique regions in the ST4 strain *C. sakazakii* 701, originally isolated from the peritoneal fluid of a fatal neonatal meningitis case.

In summary, the comparison of the draft genomes representing the *Cronobacter* genus has revealed the core genes and accessory genes unique to each strain. It was found that *C. sakazakii* has acquired the ability to use exogenous sialic acid, which may be important in the colonisation of the intestinal tract. Mobile traits such as adhesins, T6SS, and metal resistance as well as prophages have contributed considerably to the variation of genomic content and probably account for much of the variation in clinical and environmental distribution of species and sequence types. The lack of clearly identifiable virulence genes unique to *C. sakazakii* ST4 may indicate its prevalence in neonatal meningitis cases due to environmental persistence and increasing host exposure. Further improvement of the draft genome sequences, bioinformatics searches for novel or known virulence traits present in bacteria with similar modes of infection, and *in silico* and *in vitro* ascertainment of the contribution of single nucleotide polymorphisms, genome rearrangements, and other sequence features to pathogenicity may shed light on acquired pathogenicity of ST4 strains.

## Materials and Methods

### Strains and Culture Conditions

*Cronobacter* strains were selected to represent the seven recognized species, including those from reported clinical cases as well as species type strains (Table 1). All *Cronobacter* strains were stored at −80°C in Nutrient Broth (Oxoid, UK) with 10% glycerol, subcultured on Trypticase Soy Agar (Oxoid ThermoFisher, UK) and checked for purity. Overnight Trypticase Soy Broth (Oxoid ThermoFisher, UK) cultures were used for DNA extraction.

### Genome Sequencing and Assembly

Genomic DNA was isolated using the Qiagen DNeasy Blood and Tissue DNA Isolation Kit according to manufacturer’s instructions. *Cronobacter* genomes of all strains except 680, 1210^T^ and 1330^T^ were sequenced using the SOLiD™ 4 system (Life Technologies, Carlsbad, CA). Long mate-paired genomic DNA libraries with approximately 1.8 kb inserts were sequenced to generate 23–36 million of 2×50 bp reads for each strain, approximating 500–800 fold coverage of the genome. The colorspace reads were error-corrected and then assembled *de novo* into contigs and scaffolds using the Velvet assembly engine [63]. The ultimate genome assemblies contain 1,600 to 3,100 contigs with N50 of 3.7 to 5.5 kb and 260 to 1,170 scaffolds with N50 of 230 to 600 kb (Table 3).

**Table 3:** *De novo* assembly statistics for the eleven newly sequenced *Cronobacter* strains.

Species	Strain	Sequencing platform	Total cont length (bp)	N50 of scaffolds (bp)	Number of scaffolds	N50 of contigs (bp)	Number of contigs	Estimated number of ORFs
*C. sakazakii*	680	PGM	4,357,873	nd^a^	nd	51,120	194	4,178
	696	SOLiD	4,897,138	297,746	920	4,336	2,659	4,661
	701	SOLiD	4,752,729	346,235	1,171	3,538	3,148	4,509
*C. malonaticus*	507	SOLiD	4,447,701	373,979	464	3,703	2,361	4,226
	681	SOLiD	4,496,745	345,762	263	5,537	1,592	4,291
*C. turicensis*	564	SOLiD	4,500,608	411,105	263	4,796	1,807	4,227
*C. dublinensis*	582	SOLiD	4,677,592	229,230	539	3,822	2,657	4,483
	1210	PGM	4,594,228	nd	nd	46,941	210	4,376
*C. muytjensii*	530	SOLiD	4,533,101	596,924	444	4,925	1,937	4,304
*C. universalis*	NCTC 9529^T^	SOLiD	4,450,737	331,248	389	4,506	2,085	4,316
*C. condimenti*	1330	PGM	4,469,5362	nd	nd	83,159	137	4,307

a nd indicates that scaffolding was not performed due to lack of mate-paired libraries.

Strains 680, 1210^T^ and 1330^T^ were sequenced using Ion Torrent PGM system (Life Technologies). Fragment library preparation was performed with the Ion Fragment Library Kit (Life Technologies, Darmstadt, Germany). Template preparation was carried out with the Ion Xpress™ Template Kit (Life Technologies). The Ion Sequencing Kit (Life Technologies) was used with the Personal Genome Machine™ (PGM™) sequencer. A single sequencing run (65 cycles) was performed on an Ion 316™ chip for each library. Contigs were assembled from fragment reads using the MIRA 3 assembler [http://sourceforge.net/apps/mediawiki/mira-assembler/index.php].

The assembled genome scaffolds were aligned to the most closely related publicly available genomes using MUMmer [64]. The scaffolds of strains 680, 696, 701, 507, and 681^T^ were aligned to the *C. sakazakii* BAA-894 complete genome (accession numbers NC009778 - NC009780). The scaffolds of strains 564, 582, 1210^T^, 530, 581^T^ and 1330^T^ were aligned to the *C. turicensis* z3032 complete genome (accession numbers NC013282-NC013285). Scaffolds were broken at points where non-contiguous regions of the reference genome were juxtaposed and then re-ordered so that they were syntenic with the reference genome. All scaffolds from a given strain were concatenated into a single pseudogenome, separated by the sequence, NNNNCACACACTTAATTAATTAAGTGTGTGNNNNN, which contains stop codons in all six reading frames. Scaffolds that did not match the reference genomes were concatenated in random orders at the end of the genome. The pseudogenomes were annotated using the RAST automated annotation server [35].

The genome sequences of the eleven newly sequenced strains were deposited to Genbank (see Table 1 for accession numbers).

### Core and Pan Genome Identification

The genomes were compared using methods similar to those previously published [65]. Orthologous and paralogous gene families were initially constructed based on RAST annotations. An ‘all-against-all’ tblatx matrix was constructed using BLAT [66]. The blat matrix was used as an input to construct orthologous/paralogous gene families using OrthoMCL [67]. A representative nucleotide sequence from each gene family was collated with strain-specific genes not present in any gene families. This set of representative sequences was again subjected to a tblatx analysis against the whole genome nucleotide sequences to identify candidate genes that may have been missed by the annotation server RAST. A gene was considered present if it had at least 85% nucleotide sequence identity covering at least 20% of the sequence length. The phyletic table generated from the tblatx analysis was consolidated with the phyletic table generated from OrthoMCL analysis to compute a comprehensive pan genome matrix. This phyletic matrix was used as an input for the binomial mixture model software of Snipen et al. [36] to determine the *Cronobacter* core genome and accessory genes.

### Prophage Identification

ACLAME Prophinder, available at http://aclame.ulb.ac.be/Tools/Prophinder/, was used for prophage identification [34].

#### Phylogenetic analysis

Core genome analysis revealed that there were 1117 annotated gene families conserved across all genomes in exactly one copy. Orthologous DNA sequences within each gene family were aligned using Muscle [68]. Individual alignments were concatenated using custom scripts, and a maximum likelihood (ML) tree was constructed using RaxML (Ver. 7.2.6) [69]. As a distinct alternative, a majority rule consensus tree of all gene family trees that were conserved across all genomes and were present in exactly one copy was constructed using Phylip (Ver 3.69) [70]. A concatenated alignment of 100 randomly chosen loci from the 1117 single copy core loci was used as an input for Clonal Frame [33]. Two independent runs, each consisting of 200,000 Markov chain – Monte Carlo (MCMC) iterations were performed, where the first 100,000 iterations were discarded as burn-in. The runs were compared for convergence using the Gelman- Rubin statistic [71], which was found to be satisfactory.

#### Evolutionary rate of gene families

Individual gene sequences (DNA and amino acid sequences) for all genes in gene families predicted by OrthoMCL were extracted using custom Perl scripts. Codon alignments within gene families were constructed using PAL2NAL [72]. Amino acid alignments needed as input for PAL2NAL were constructed using Muscle [68]. The dS/dN ratios for all possible pairwise comparisons of the codon alignments within a gene family were calculated using SNAP [73]. Mean dS/dN ratios were assigned for individual gene families by averaging all pairwise ratios within each family. The pan genome was ranked based on logarithmic (log_10_) dS/dN ratios; enrichment analysis for gene sets containing relatively fast evolving gene families was conducted using Gene Set Enrichment Analysis (GSEA) [74]. Within GSEA, controlled false discovery rates were used to adjust p values for multiple comparisons.

### Multilocus Sequence Analysis (MLSA)

MLSA was undertaken by using existing sequences in the multilocus sequence typing (MLST) database (http://www.pubMLST.org/cronobacter) and for new ones obtained in this study. Multilocus sequence typing of strains was performed as previously described [17]. The sequences obtained were independently aligned with sequences of the type strains of all species of the genus *Cronobacter* using the ClustalW2 program [75] and MEGA (Molecular Evolutionary Genetics Analysis) software version 5 [76]. Genetic distances and clustering were determined using Kimura’s two-parameter model [77]; evolutionary trees were reconstructed by the Maximum Likelihood method [78]. Stability of the relationships was assessed by the bootstrapping method (1000 replicates).

## Supporting Information

Figure S1 **BLAST Ring Image Generator (BRIG) analysis of the** ***Cronobacter*** **genomes.**
(DOC)

Figure S2 **BLAST Ring Image Generator (BRIG) analysis of** ***Cronobacter*** **plasmid pESA3 with matching sequence content found in other** ***Cronobacter*** **species.**
(DOC)

Figure S3 **BLAST Ring Image Generator (BRIG) analysis of** ***Cronobacter*** **plasmid pCTU1 to matching content in other** ***Cronobacter*** **species.**
(DOC)

Table S1 **Number of putative prophage regions in the** ***Cronobacter*** **genomes.**
(DOC)

Table S2 **Presence (+) or absence (−) of type six secretion systems in** ***Cronobacter*** **spp.**
(DOC)

Table S3 **Copy number variation of the hemolysin gene within the** ***Cronobacter*** **genus.**
(DOC)

Text S1 **Additional regions of variation between** ***Cronobacter*** **genomes.**
(DOC)
