# Supplementary material for: Comparative Analysis of Genome Sequences Covering the Seven Cronobacter Species
Source: PLoS One. 2012 Nov 16;7(11):e49455. doi: 10.1371/journal.pone.0049455 (PMC3500316; doi:10.1371/journal.pone.0049455)

Figure S1. BLAST Ring Image Generator (BRIG) analysis of the *Cronobacter* genomes using the *C. sakazakii* BAA-894 genome as a reference backbone.


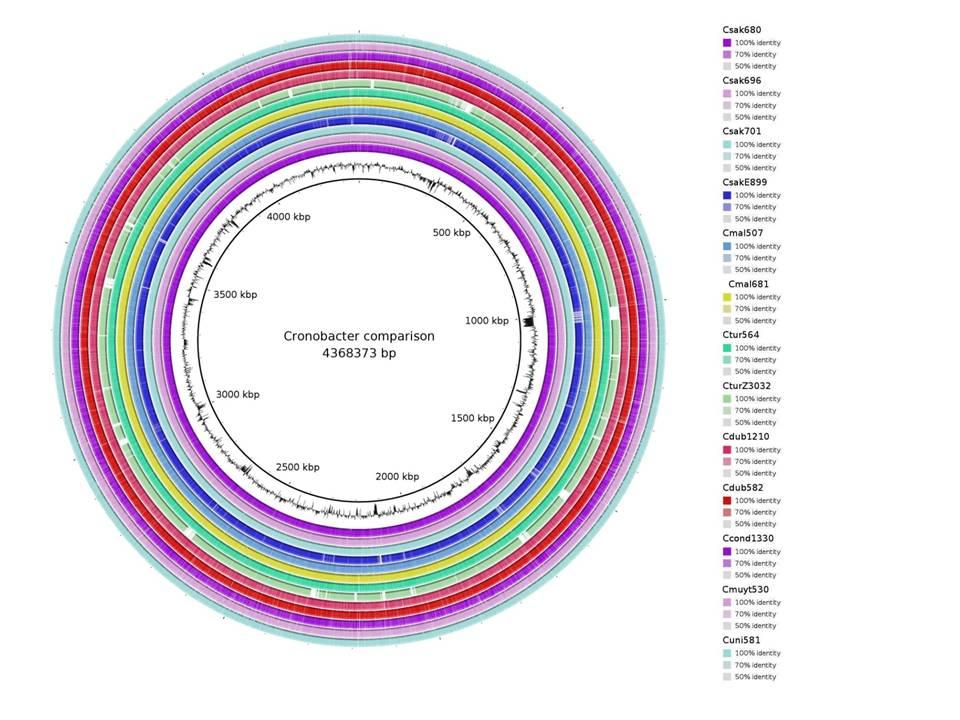

Supplement: Figure S1 — BLAST Ring Image Generator (BRIG) analysis of the Cronobacter genomes. (DOC) [file pone.0049455.s001.doc]
