# Supplementary material for: Comparative Analysis of Genome Sequences Covering the Seven Cronobacter Species
Source: PLoS One. 2012 Nov 16;7(11):e49455. doi: 10.1371/journal.pone.0049455 (PMC3500316; doi:10.1371/journal.pone.0049455)
Supplement: Figure S2 — BLAST Ring Image Generator (BRIG) analysis of Cronobacter plasmid pESA3 with matching sequence content found in other Cronobacter species. (DOC) [file pone.0049455.s002.doc]

Figure S2. BLAST Ring Image Generator (BRIG) analysis of *Cronobacter* plasmid pESA3 with matching sequence content found in other *Cronobacter* species.


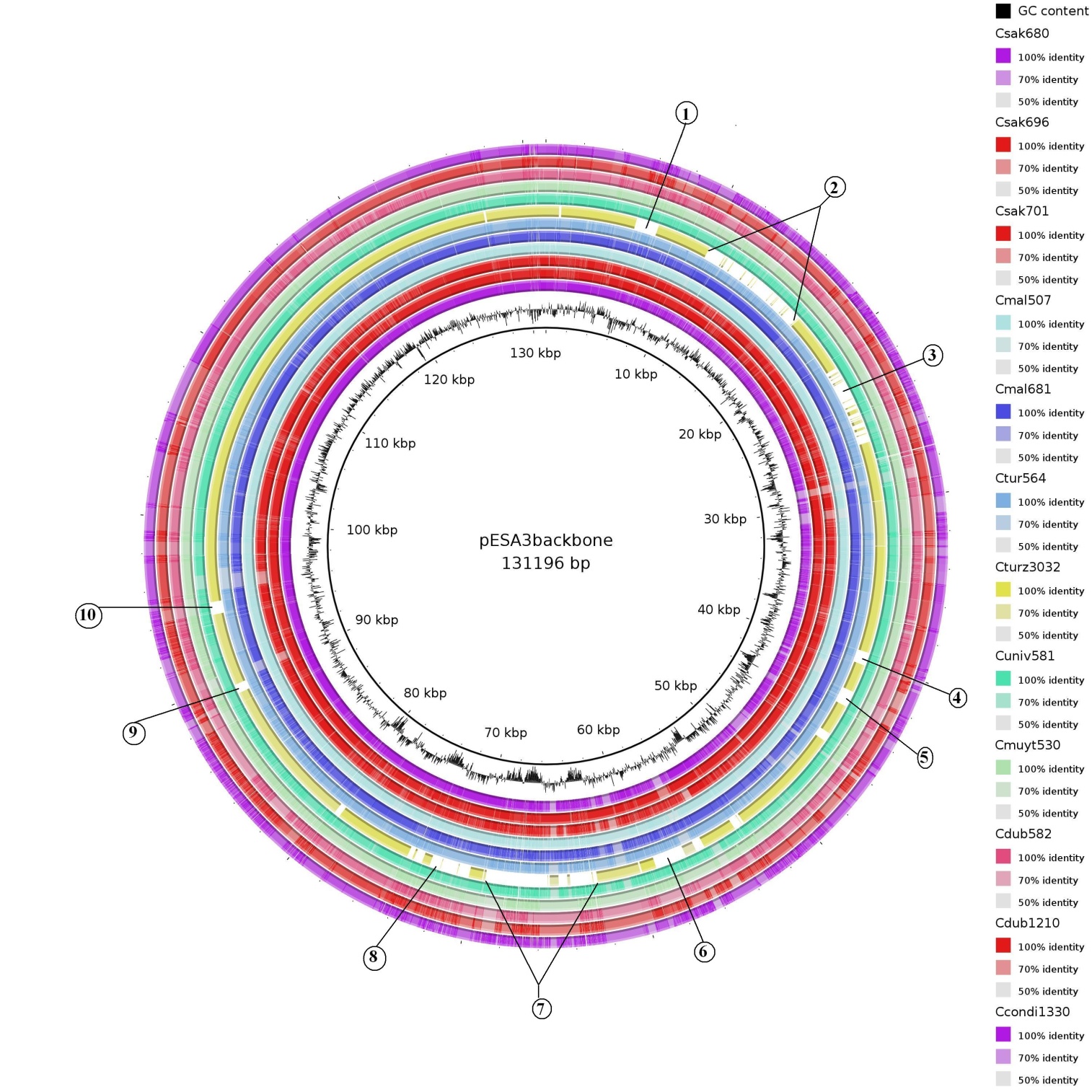


| Region | Locus | Annotation |
| --- | --- | --- |
| 1 | ESA_pESA3p05434 | Plasminogen activator Pla (EC 3.4.23.48). Aspartic peptidase. MEROPS family A26 (EC:3.4.23.49) |
|  | ESA_pESA3p05435 | Hypothetical protein - CpmJ protein |
| 2 | ESA_pESA3p05440 | Outer membrane protein V |
|  | ESA_pESA3p05441 | Response regulators consisting of a CheY-like receiver domain and a winged-helix DNA-binding domain |
|  | ESA_pESA3p05442 | Signal transduction histidine kinase |
|  | ESA_pESA3p05443 | RND family efflux transporter, MFP subunit |
|  | ESA_pESA3p05444 | Cation/multidrug efflux pump |
| 3 | ESA_pESA3p05451 | ABC-type molybdate transport system, periplasmic component |
|  | ESA_pESA3p05452 | Transcriptional regulator, LysR family |
| 4 | ESA_pESA3p05478 | Diguanylate cyclase (GGDEF) domain |
| 5 | ESA_pESA3p05480 | Hypothetical protein |
|  | ESA_pESA3p05481 | Type VI secretion system effector, Hemolysin co-regulated protein1 family |
| 6 | ESA_pESA3p05494 | Type IV / VI secretion system protein, DotU family |
|  | ESA_pESA3p05495 | Outer membrane protein and related peptidoglycan-associated (lipo)proteins |
| 7 | ESA_pESA3p05498 | Hypothetical protein |
|  | ESA_pESA3p05499 | Hypothetical protein |
|  | ESA_pESA3p05500 | Type VI secretion system Vgr family protein |
|  | ESA_pESA3p05501 | Hypothetical protein |
|  | ESA_pESA3p05502 | Hypothetical protein |
|  | ESA_pESA3p05503 | Hypothetical protein |
|  | ESA_pESA3p05504 | Uncharacterized conserved protein |
|  | ESA_pESA3p05505 | Hypothetical protein |
|  | ESA_pESA3p05506 | Type VI secretion lipoprotein, VC_A0113 family |
| 8 | ESA_pESA3p05508 | Thiol:disulfide interchange protein/Uncharacterized protein predicted to be involved in C-type cytochrome biogenesis/ suppressor for copper-sensitivity ScsB |
|  | ESA_pESA3p05509 | Suppression of copper sensitivity: putative copper binding protein ScsA |
| 9 | ESA_pESA3p05526 | Hypothetical protein |
| 10 | ESA_pESA3p05530 | Glutathione S-transferase (EC:2.5.1.18) |
