# Supplementary material for: Comparative Analysis of Genome Sequences Covering the Seven Cronobacter Species
Source: PLoS One. 2012 Nov 16;7(11):e49455. doi: 10.1371/journal.pone.0049455 (PMC3500316; doi:10.1371/journal.pone.0049455)
Supplement: Figure S3 — BLAST Ring Image Generator (BRIG) analysis of Cronobacter plasmid pCTU1 to matching content in other Cronobacter species. (DOC) [file pone.0049455.s003.doc]

Figure S3. BLAST Ring Image Generator (BRIG) analysis of *Cronobacter* plasmid pCTU1 to matching content found in other *Cronobacter* species.


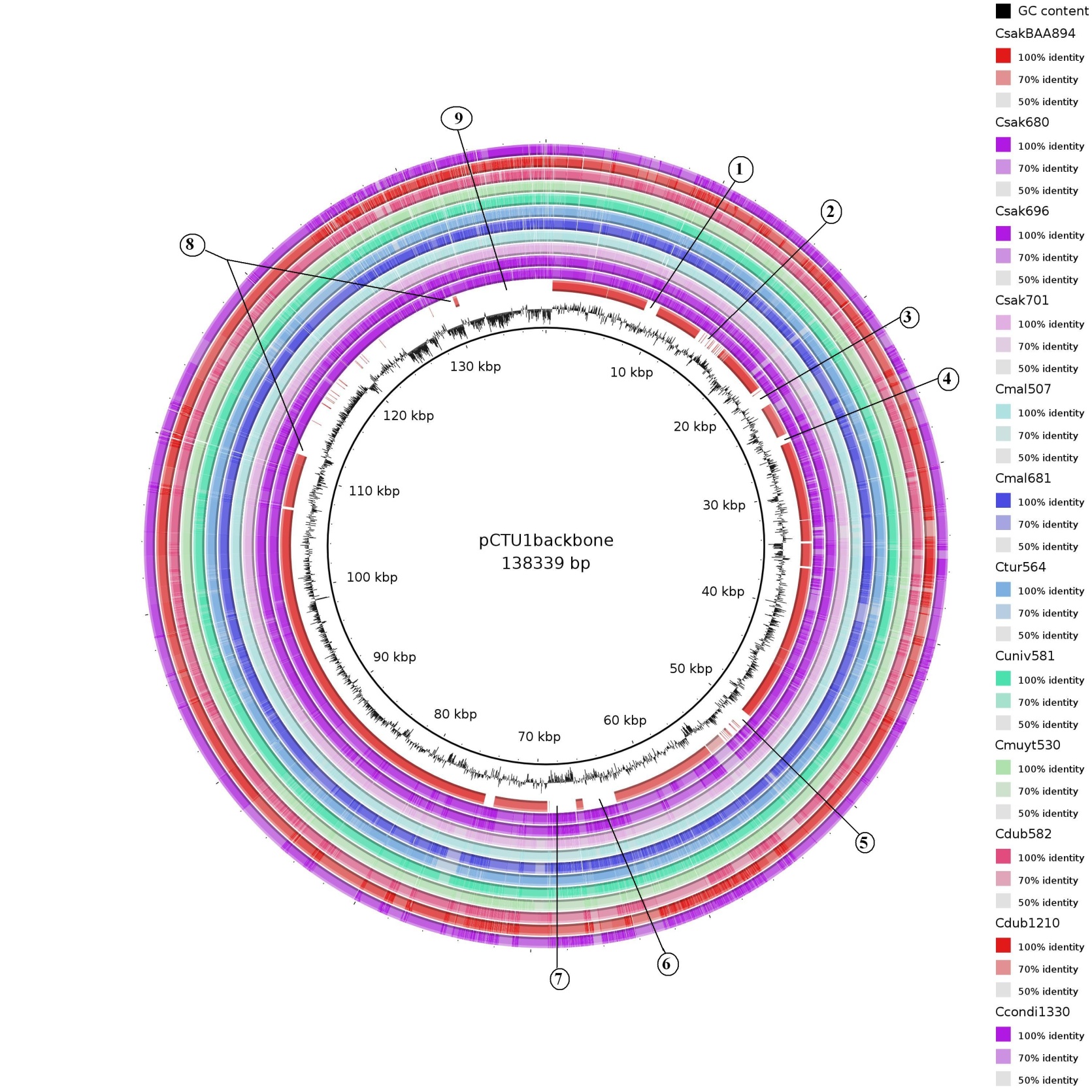


| Region | Locus | Annotation |
| --- | --- | --- |
| 1 | Ctu_1p00130 | Hypothetical protein, ygjN |
|  | Ctu_1p00140 | HTH-type transcriptional regulator, ygjM |
| 2 | Ctu_1p00210 | Transcriptional regulator, LysR family |
|  | Ctu_1p00220 | Permeases of the major facilitator superfamily |
| 3 | Ctu_1p00320 | Tellurite resistance protein, TehA |
| 4 | Ctu_1p00360 | Thiol:disulfide interchange protein, DsbC |
| 5 | Ctu_1p00640 | Beta-glucoside bgl operon antiterminator, BglG family |
| 6 | Ctu_1p00740 | hypothetical protein |
|  | Ctu_1p00750 | hypothetical protein |
| 7 | Ctu_1p00780 | hypothetical protein |
|  | Ctu_1p00790 | hypothetical protein |
| 8 | Ctu_1p01150 | Two partner secretion protein/Hemolysin activator protein precursor, fhaC |
|  | Ctu_1p01160 | fhaB |
|  | Ctu_1p01170 - Ctu_1p01250 | hypothetical proteins |
| 9 | Ctu_1p01270 - Ctu_1p01360 | hypothetical proteins |
