# Supplementary material for: Comparative Analysis of Genome Sequences Covering the Seven Cronobacter Species
Source: PLoS One. 2012 Nov 16;7(11):e49455. doi: 10.1371/journal.pone.0049455 (PMC3500316; doi:10.1371/journal.pone.0049455)
Supplement: Table S1 — Number of putative prophage regions in the Cronobacter genomes. (DOC) [file pone.0049455.s004.doc]

Table S1. Number of putative prophage regions in the *Cronobacter* genomes

| Species | Strain | Major Phage Regions | Minor Phage Regions |
| --- | --- | --- | --- |
| *C. sakazakii* | 658 | 3 | 3 |
|  | 680 | 2 | 8 |
|  | 696 | 2 | 11 |
|  | 701 | 4 | 5 |
| *C. malonaticus* | 507 | 2 | 4 |
|  | 681 | 1 | 10 |
| *C. turicensis* | 564 | 2 | 7 |
| *C. universalis* | 581 | 1 | 3 |
| *C. muytjensii* | 530 | 2 | 8 |
| *C. dublinensis* | 582 | 3 | 4 |
|  | 1210 | 2 | 10 |
| *C. condimenti* | 1330 | 2 | 3 |
