# Supplementary material for: Comparative Analysis of Genome Sequences Covering the Seven Cronobacter Species
Source: PLoS One. 2012 Nov 16;7(11):e49455. doi: 10.1371/journal.pone.0049455 (PMC3500316; doi:10.1371/journal.pone.0049455)
Supplement: Table S2 — Presence (+) or absence (−) of type six secretion systems in Cronobacter spp. (DOC) [file pone.0049455.s005.doc]

Table S2. Presence (+) or absence (-) of type six secretion systems in *Cronobacter* spp.

| Cluster |  | Csak_BAA894 | Csak_701 | Csak_E899 | Csak_680 | Csak_696 | Cmal_507 | Cmal_681 | Ctur_564 | Ctur_Z3021 | Cuniversalis_581 | Cmuyt_530 | Cdub_582 | Cdub_1210 | Ccondimenti_1330 | Comments |
| --- | --- | --- | --- | --- | --- | --- | --- | --- | --- | --- | --- | --- | --- | --- | --- | --- |
|  |  |  |  |  |  |  |  |  |  |  |  |  |  |  |  |  |
| 1 | ESA_00140–ESA_00145 | + | + | + | + | + | + | - | + | + | + | + | + | + | + | Encodes most of the proteins that are conserved across different T6SS clusters, including DotU homologue (ESA_00140), Vgr homologue (ESA_00141), and a putative lipoprotein from the VC_A0113 family (ESA_00145) |
|  |  |  |  |  |  |  |  |  |  |  |  |  |  |  |  |  |
| 2 | ESA_02035–ESA_02040 | + | + | + | - | - | - | - | + | + | + | + | - | - | - | Includes Vgr-type protein (ESA_02035), a lipoprotein from the VC_A0113 family (ESA_02038), and other genes homologous to proteins encoded in the T6SS clusters |
|  |  |  |  |  |  |  |  |  |  |  |  |  |  |  |  |  |
| 3 | ESA_02735–ESA_02740 | + | + | - | - | + | + | - | - | + | - | - | + | + | - | Contains genes encoding SciE-type protein (ESA_02736), Vgr-type protein (ESA_02739), and a protein homologous to phage gp7 protein; adjacent to phage |
|  |  |  |  |  |  |  |  |  |  |  |  |  |  |  |  |  |
| 4 | ESA_03887–ESA_03946 | + | + | + | + | + | + | + | + | + | + | + | + | + | + | Longest and most complete cluster; included genes encoding Vgr-type proteins (ESA_03905 and ESA_03917), IcmF-type protein (ESA_03945), DotU-type protein (ESA_03946), ClpV ATPase (ESA_03921), SciE-type protein (ESA_03925), Ser/Thr protein phosphatase (ESA_03927), and Ser/Thr protein kinase (ESA_03920) |
|  |  |  |  |  |  |  |  |  |  |  |  |  |  |  |  |  |
| 5 | ESA_pESA3p05491–ESA_pESA3p05506 | + | - | - | + | + | - | - | - | - | - | - | + | - | - | Plasmid borne; ESA_pESA3p05494 encodes DotU-like protein and ESA_pESA3p05495 encodes a protein with a C-terminal extension with similarity to ompA. ESA_pESA3p05497 encodes a ClpV ATPase and ESA_pESA3p05500 encodes a Vgr-like protein. |
|  |  |  |  |  |  |  |  |  |  |  |  |  |  |  |  |  |
| 6 | Ctu_12090-Ctu_12210 | - | + | - | - | + | + | - | - | + | - | - | + | + | - |  |
