# Supplementary material for: Comparative Analysis of Genome Sequences Covering the Seven Cronobacter Species
Source: PLoS One. 2012 Nov 16;7(11):e49455. doi: 10.1371/journal.pone.0049455 (PMC3500316; doi:10.1371/journal.pone.0049455)
Supplement: Table S3 — Copy number variation of the hemolysin gene within the Cronobacter genus. (DOC) [file pone.0049455.s006.doc]

Table S3. Copy number variation of the hemolysin gene within the *Cronobacter* genus

| Loci | Description | Csak_BAA894 | Csak_701 | Csak_E899 | Csak_680 | Csak_696 | Cmal_507 | Cmal_681 | Ctur_564 | Ctur_Z3021 | Cuniversalis_581 | Cmuyt_530 | Cdub_582 | Cdub_1210 | Ccondimenti_1330 |
| --- | --- | --- | --- | --- | --- | --- | --- | --- | --- | --- | --- | --- | --- | --- | --- |
| ESA_00102 | Hemolysin activator protein precursor | 1 | 1 | 1 | 2 | 1 | 0 | 2 | 2 | 2 | 2 | 2 | 2 | 3 | 3 |
| ESA_00432 | COG1272: Predicted membrane protein hemolysin III homolog | 1 | 1 | 1 | 1 | 1 | 1 | 1 | 1 | 1 | 1 | 1 | 1 | 1 | 1 |
| ESA_00643 | Hemolysins and related proteins containing CBS domains | 1 | 1 | 1 | 0 | 1 | 1 | 1 | 1 | 1 | 1 | 1 | 1 | 1 | 1 |
| ESA_02810 | Hemolysin expression modulating protein | 1 | 1 | 1 | 1 | 1 | 1 | 1 | 1 | 1 | 1 | 1 | 1 | 1 | 1 |
| ESA_02937 | Putative hemolysin (smaller - ~1790 bp) | 1 | 1 | 1 | 1 | 1 | 1 | 1 | 1 | 1 | 1 | 0 | 0 | 0 | 0 |
|  | Hemolysin (~2200 bp) | 0 | 0 | 0 | 0 | 0 | 0 | 1 | 0 | 2 | 1 | 1 | 1 | 2 | 1 |
| ESA_03540 | 21 kDa hemolysin precursor | 1 | 0 | 1 | 1 | 1 | 0 | 1 | 1 | 1 | 1 | 1 | 1 | 1 | 1 |
|  | Possible adhesin/hemolysin precursor | 0 | 0 | 0 | 1 | 0 | 0 | 1 | 0 | 1 | 0 | 1 | 1 | 0 | 0 |
