# Supplementary material for: Comparative Analysis of Genome Sequences Covering the Seven Cronobacter Species
Source: PLoS One. 2012 Nov 16;7(11):e49455. doi: 10.1371/journal.pone.0049455 (PMC3500316; doi:10.1371/journal.pone.0049455)
Supplement: Text S1 — Additional regions of variation between Cronobacter genomes. (DOC) [file pone.0049455.s007.doc]

**Supplementary Text**

**Additional regions of variation between *Cronobacter* genomes**

*Cronobacter* plasmid content.

Only partial regions of pCTU3 (53.8 kb) were found in *C. sakazakii* strains 680, 696, 701, *C. malonaticus* 681, *C. muytjensii* 530, and *C. dublinensis* 582. No pCTU3 regions were found in *C. malonaticus* 507, *C. turicensis* 564, *C. dublinensis* 1210, *C. universalis* 581, or *C. condimenti* 1330. No genes of pESA2 (31kb) or pCTU2 (22.5kb) were found in the newly sequenced genomes, except for a small region in *C. sakazakii* 696 (p06563-700) encoding 8 ORFs, one of which was TnpA transposase. The read coverage of the *C. sakazakii* 680 genome indicates that the plasmid also encodes for a *lac* operon, iron-dicitrate transporter (*fecRABCDE*) and arsenate resistance. These traits were absent from the other sequenced strains. In contrast, the FHA loci of pCTU1 was located on the chromosome of *C. sakazakii* 680, indicating the mobility of plasmid-related traits within the *Cronobacter* genome. *C. sakazakii* 701 (ST4) contains a type VI secretion system composed of 6 genes, which is distinct from that on pESA3. This requires further analysis due the clinical association of ST4 strains with neonatal meningitis.

The variation in plasmid content extends our previous CGH studies (1) and complements others who reported the larger *Cronobacter* plasmids were based on the incompatibility group RepFIB (2). Kucerova et al. (1) reported that pESA2 was absent in all strains except *C. turicensis* which had 19 (61.3%) genes present, and *C. sakazakii* 696 which had 4 (12.9%) genes present. While plasmid profiling showed *C. sakazakii* strains ATCC 12868 (ST3), 696 (ST12) and ST4 strains 20, 701 & 767 contained a plasmid of size similar to pESA3, one strain (*C. sakazakii* ATCC 29544^T^, ST8) had a slightly shorter (110 kb) plasmid. The latter was also true for *C. malonaticus* (LMG 23826^T^). No plasmids were visible in *C. turicensis* (LMG 23827^T^), *C. dublinensis* (LMG 23823^T^) or *C. muytjensii* (ATCC 51329^T^). The percentage of pESA3 genes present in the strains with the larger plasmid (110-131 kb) varied from 44 - 89%. It was notable that *C. turicensis* LMG 23827^T^ and *C. dublinensis* LMG 23823^T^ (strains without any detectable plasmid) contained 30% and 16% of the pESA3 genes infering their chromosomal location in those species.

*Cronobacter* genome prophage content

In this study about 35% of the unique *C. sakazakii* ORFs belonged to 12 different putative prophage regions, and most of the prophage regions were shared between the genomes of *C. sakazakii* 696 and 701 (Supplementary Table S2). There was one major prophage region unique to *C. sakazakii* 680 and BAA-894 (ESA_02304-39), the latter previously characterised in detail by Kucerova et al (1) as GR10. Apart from the phage assembly proteins, these regions included a number of genes related to restriction-modification systems and DNA repair proteins. Between the two *C. malonaticus* genomes, 17 putative prophage regions were identified, three of which appeared to be intact phage genomes, whereas the rest were remnants or artefacts. One of these regions was found to be unique to the genome of strain 507, while the rest were shared with the other species. The *C. turicensis* 564 genome showed the presence of 9 putative prophage regions, only two of which were major intact prophages. They were both shared with other *Cronobacter* species. There were two unique prophage regions identified in the *C. turicensis* z3032 genome which had not been reported previously (3). About 15% of the unique ORFs in *C. dublinensis* were putative prophage regions, including a prophage region unique to *C. dublinensis* 1210. The remaining phage related regions were also found within the *C. sakazakii* genomes. The *C. universalis* genome indicated the presence of a large prophage region and some smaller artefacts. One of these regions was unique to *C. universalis*, while the rest were shared with *C. sakazakii* and *C. muytjensii* genomes. Ten putative prophage regions were identified in *C. muytjensii* 530 and are shared with the genomes of *C. sakazakii, C. universalis* and *C. dublinensis*. There were five putative prophage regions in *C. condimenti* strain 1330, of which two appeared to be large intact genomes. One phage region was unique to *C. condimenti*, while the other regions were shared with the *C. sakazakii* genomes.

*Cronobacter* genome variation

Regions of genome variation with *C. sakazakii* included genes involved in Type I restriction-modification systems, Psi operon genes, tellurium (*Ter* gene cluster) and a large region containing *Tra* genes belonging to the Inc plasmid family. The genome of *C. sakazakii* BAA-894 showed unique regions containing genes for tellurite resistance (ESA_01796-801) and some Type I fimbrial assembly proteins (ESA_01970-76). These regions had previously been highlighted in CGH studies (1,4).

*C. sakazakii* region (ESA_03609–13) encodes for the uptake and utilization of exogenous sialic acid. Five enzymes are required to catabolize N-acetylneuraminic acid (Neu5Ac), the most commonly found sialic acid. Neu5Ac lyase (NanA) breaks down Neu5Ac into N-acetylmannosamine (ManNAc) and phosphoenolpyruvate (PEP). NanK is an ATP-dependent kinase specific for ManNAc generating N-acetylmannosamine-6-phosphate (ManNAc-6-P). ManNAc-6-P epimerase (NanE) converts ManNAc-6-P into N-acetylglucosamine-6-P (GlcNAc- 6-P). GlcNAc-6-P deacetylase (NagA) and glucosamine-6-P deaminase (NagB) converts GlcNAc-6-P into fructose-6-P (Fru-6-P), which is a substrate in the glucolytic pathway. The genes for the first three enzymes (NanA, NanK and NanE) are usually found clustered together forming the *Nan* cluster. The genes encoding NagA and NagB vary in their locations among the different genomes that encode the *Nan* cluster.

Variation in O-antigen

In addition to ESA_01179-89, a second region, ESA_04101–09, also encodes for genes in O-polysaccharide biogenesis and varies across the genus. ESA_04102 encodes a glycosyltransferase involved in cell wall biogenesis and was present in all *Cronobacter* strains, whereas a putative O-antigen ligase glycosyltransferase (ESA_04103) and lipopolysaccharide heptosyltransferase III (ESA_04105) were only present in *C. sakazakii*.

In *C. sakazakii* BAA-894 (ST1) (5) LPS is a branched polymer of pentasaccharide units composed of 2-acetamido-2-deoxy-D-galactose, 3-(N-acetyl-L-alanylamido)-3-deoxy-D-quinovose, D-glucuronic acid, and D-glucose. *C. sakazakii* strain 767 (ST4) is also a branched polymer but of a repeating heptasaccharides composed of 2-acetamido-2-deoxy-D-glucose, D-galacturonic acid, L-rhamnose, and D-glucose (6). *C. malonaticus* LPS (54) is also a branched pentasaccharide unit of 2-amino-2-deoxy-D-glucose, 2-amino-2-deoxy-D-galactose, 3-deoxy-D-manno-oct-2-ulosonic acid, D-galactose and D-glucose residues. In turn, *C. muytjensii* LPS (7) is a linear unbranched pentasccharide polymer of 2-acetamido-2-deoxy-D-galactose, 2-acetamido-2-deoxy-D-glucose, 2-acetamido-3-deoxy-D-quinovose, L-rhamnose and D-glucuronic acid. These considerable differences correspond with the lack of sequence conservation as revealed in the genomic comparison. The individual genes encoding some of the differences in enzymology have yet to be elucidated.

Distribution of *Cronobacter* virulence associated genes

Many bacterial species exploit specialized secretion systems to transfer macromolecules across their membranes, either into the extracellular environment or directly into target cells. Hence, these can be important pathogenicity traits. The secretory systems are distinguishable by their conserved structural components, the secreted effectors and the secretion mechanism. The type II, type V and two partner secretion systems (T2SS, T5SS and TPS) mediate protein secretion in two steps; a Sec-dependent transport of effectors into the periplasm followed by a distinct contiguous step through the outer membrane. This differs from type I, type III and most type IV secretion systems (T1SS, T3SS and most T4SS) in which effectors are transported in a single step through both the inner and outer membranes (8). *Cronobacter* lacks Type III and V secretion systems but does contain Type IV and VI depending on strain and species. The presence of genes associated with Type VI secretion systems are given Supplementary Table S3.

Superoxide dismutase activity has been proposed as one of the mechanisms for macrophage survival in *Cronobacter* (9). However, all *Cronobacter* strains contained *sodA* homologues and therefore there is no correlation between *sodA* and the variance in virulence with species or sequence type. One macrophage infectivity potentiator-related protein (ESA_01868) was found in all *C. sakazakii*, *C. malonaticus*, *C. turicensis*, *C. muytjensii* and *C. condimenti* strains but was absent from *C. universalis* and *C. dublinensis*. This requires further analysis but does correlate with the absence of *C. universalis* and *C. dublinensis* in clinical isolates.

A complete ABC-type multidrug efflux system (ESA_01116–19) was only found in *C. sakazakii*. It comprised an outer membrane efflux protein from a family that includes TolC, a permease component (ESA_01118) of the ABC type system, and an ATPase component (ESA_01119) of the efflux system. A diverse group of proteins (ESA_02125–29) for which no common assignment to a pathway or mechanism could be found was detected in *C. sakazakii* and *C. malonaticus* only. The region includes acetyltransferases, a transcriptional regulator from the lysR family, and a putative esterase/lipase. ESA_02129 encodes a serine protease inhibitor ecotin. Ecotins have been shown to protect bacteria against the effects of neutrophil elastase (10). Another complete ABC multidrug transport system may be encoded at loci ESA_02549–53 which was present in all *Cronobacter* species except *C. muytjensii*.
